# Role of quaternary epitopes in eliciting broad neutralization response against multiple flaviviruses

**DOI:** 10.1128/jvi.01777-25

**Published:** 2026-06-15

**Authors:** Ananya Chatterjee, Vidya Mangala Prasad

**Affiliations:** 1 Molecular Biophysics Unit, Indian Institute of Science, Bangalore, India; 2 Center for Infectious Diseases Research, Indian Institute of Science, Bangalore, India; Indiana University Bloomington, Bloomington, Indiana, USA

**Keywords:** broadly neutralizing antibodies, quaternary epitopes, flaviviruses, vaccine, structural biology

## Abstract

Flaviviruses, or orthoflaviviruses, are arthropod-borne RNA viruses that pose a significant global health threat and co-circulate in many regions across the world. Neutralizing antibodies against specific pathogenic flaviviruses, such as dengue virus, Zika virus, and West Nile virus, are well-known. However, vaccine and therapeutic development against many flaviviruses remains challenging due to their antigenic diversity and complex humoral responses. Recent studies have reported patient-derived neutralizing antibodies that are active against multiple flaviviruses. Structural characterization of these pan-flavivirus antibodies has identified quaternary epitopes that are conserved across multiple flaviviruses, emphasizing the importance of quaternary epitopes on native flaviviruses, which, in some cases, are also preserved across different virus morphologies. Understanding the basis of quaternary structural epitope recognition can aid in the design of immunogens that elicit broad protection, with a lower risk of infection enhancement. In this review, we discuss mechanistic insights of bnAbs targeting different quaternary epitopes found on pathogenic flaviviruses, and implications for next-generation pan-flavivirus vaccine strategies.

## INTRODUCTION

Flaviviruses, or orthoflaviviruses (*flavus* in Latin meaning yellow because of jaundice induced by the yellow fever virus) (1, 2), are arthropod-borne RNA viruses that cause severe illness in humans. The flavivirus genus comprises medically important viruses, including dengue (DENV), yellow fever virus (YFV), Zika virus (ZIKV), West Nile virus (WNV), Japanese encephalitis virus (JEV), Kyasanur forest disease virus (KFDV), and tick-borne encephalitis virus (TBEV), that pose significant global health threats (3). It is estimated that 400 million flavivirus infections occur worldwide each year (4, 5).

The canonical flavivirion is a spherical particle, approximately 50 nm in diameter, that displays an icosahedral arrangement of its surface glycoproteins. The viral RNA genome is a positive-sense, single-stranded molecule of ~11 kb in size and is enclosed by the capsid protein (~12 kDa) to form the nucleocapsid. The nucleocapsid is surrounded by a host-derived lipid membrane, whose external surface is densely decorated with two proteins, the envelope glycoprotein (E) (~53 kDa) and membrane protein (M) (~8 kDa). The E-protein is the only surface-exposed protein on the virus and is necessary for cellular entry via receptor-mediated endocytosis and membrane fusion (6). The E-protein is also an essential antigenic determinant and a crucial target for inducing neutralizing antibodies (7).

**Fig 1:**
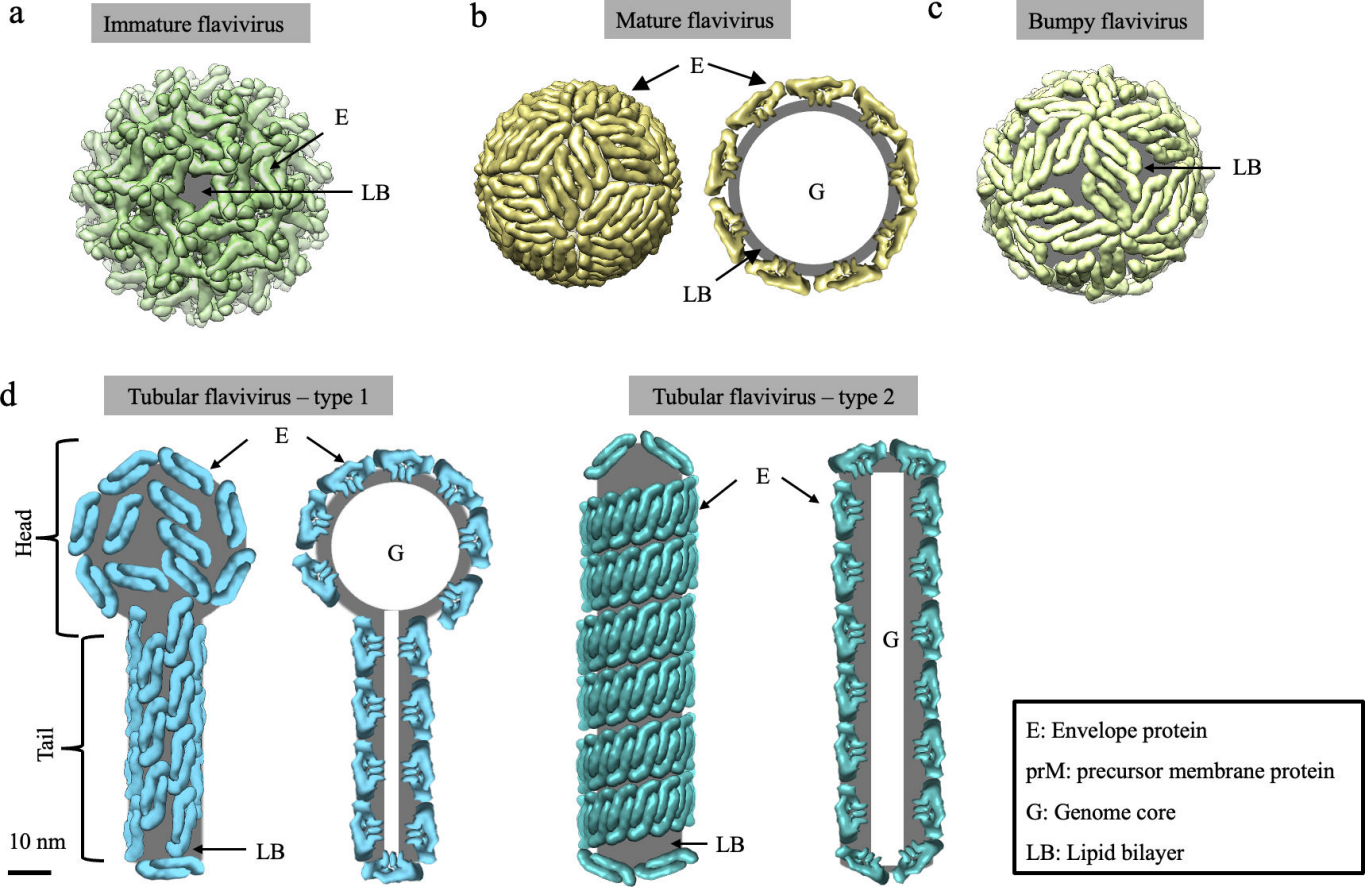
Different morphologies observed in flaviviruses. Surface representation of (**a**) immature virus, (**b**) mature virus and its cross-section, (**c**) bumpy virus, and (**d**) tubular virus type 1 and type 2. Envelope protein: E, prM: precursor membrane protein, LB: lipid bilayer, G: genome core. Spherical viruses are shown in shades of green, and tubular viruses are shown in shades of blue. The scale bar indicates 10 nm.

Flavivirus’s replication cycle starts with the virus cell entry via interaction with multiple types of cell attachment factors or receptors (glycosaminoglycans, DC-SIGN, heparan sulfate, mannose receptor, and TIM-1), followed by receptor-mediated endocytosis and fusion in the low pH conditions of late endosomes (8, 9). These steps are orchestrated by conformational changes in the viral E-protein. Genome release after viral membrane fusion leads to viral genome replication and protein production in the endoplasmic reticulum (ER). The viral RNA genome is translated into a polyprotein comprising three structural proteins (C, Precursor M [prM], and E proteins) along with seven non-structural proteins (3, 10, 11). The viral nucleocapsid buds into the ER lumen and acquires a membrane surface that contains the E protein and prM protein as 60 trimeric spikes (prM_3_E_3_). The assembled virions are then transported via the trans-Golgi network, where massive conformational changes occur in the virus morphology. The immature virus (Fig. 1), displaying prM_3_E_3_ surface spikes, rearranges into a relatively smooth lattice of prM-E dimers, which is followed by furin cleavage of the prM protein, separating the pr peptide from the M protein. After cleavage, the pr protein still caps the E protein and protects the viral fusion loop. Once the virus exits the cell via exocytosis, in the neutral pH of the extracellular space, pr is separated from E, and the fusion loop is primed, thereby generating a mature, infectious virion (Fig. 1) (12, 13). The mature virion displays 90 non-covalently bound, antiparallel E-M dimers (M_2_E_2_) on the virus surface in a herringbone pattern (Fig. 1) (14). However, this maturation process is not always complete and can result in the production of a mixed population comprising completely mature, partially mature, and immature virus particles. These partially mature particles can be infectious and have important implications for antibody recognition, neutralization, and enhancement during flavivirus infection (15–18). Although immature particles lack the ability to infect cells, it has been shown that fully immature virus particles can also potentially become infectious upon interacting with anti-prM antibodies from prior infections, as reported in the case of DENV (19, 20).

In the past decade, other morphologies have also been reported in certain flaviviruses. DENV has been reported to exist as expanded/bumpy viruses when incubated at 37°C–40°C (21–24), whereas some strains of both DENV and ZIKV have exhibited tubular forms of the virions (Fig. 1) (25–27). Different strains of flaviviruses demonstrate varying tendencies to form these alternate morphologies, a phenomenon that has been previously reviewed (23, 24) but not yet completely understood.

## IMMUNE RESPONSE AGAINST FLAVIVIRUSES

In the flavivirus genus, almost all viruses are treated as single species having different strains or genotypes (e.g., ZIKV, JEV, YFV, and WNV) (5, 28). Only DENV has defined serotypes (I–IV) that are antigenically distinct with ~65% genetic similarity, and each serotype consists of multiple strains (29). Thus, upon infection with one DENV serotype, a patient remains immune to subsequent infection with the same serotype, but upon further infection with other serotypes, neutralizing antibodies that were generated against an earlier serotype might not be effective. Secondary infection with a heterologous serotype, thus, can increase the risk of severe infection due to cross-reactive, but poorly neutralizing antibodies, as these antibodies can bind the virus and promote cellular entry of the virus-antibody complex via Fc gamma receptors (FcγR) (30, 31). The similar structural architecture of flaviviruses also results in cross-virus reactive antibodies in certain cases, such as between DENV and ZIKV (28). Hence, in the case of flaviviruses, antibodies play a crucial role in determining the disease outcome, as not all antibodies provide protection and, in some instances, cause antibody-dependent enhancement (ADE) and increased viremia (30, 31). In addition to the E-protein, other proteins such as prM/M and non-structural proteins like NS1, NS3, and NS5 have also been shown to be antigenic targets in flaviviruses (32, 33). Specifically, antibodies against prM facilitate the cellular entry of immature virus particles through Fc receptor-mediated endocytosis and can cause ADE (19, 20). For the purpose of this review, the focus will be on antibodies against the viral E-glycoprotein.

Monoclonal antibodies (mAbs) generated against flaviviruses can be classified into four types (34, 35). Antibodies specific toward a particular viral strain are referred to as Strain-Specific (SS), which may fail to neutralize heterologous strains within the same virus serotype. The second type is Type-Specific (TS) mAbs that can recognize antigenic determinants shared across multiple strains of a single flavivirus. The third type is Complex-reactive (CR) mAbs that can cross-neutralize flaviviruses within the same serocomplex. Flavivirus serocomplexes are classified based on shared antigenic determinants among phylogenetically related viruses, such as the DENV serocomplex, which includes all four serotypes of DENV (1–4). The final type is the Group reactive (GR) mAbs, which can bind multiple flaviviruses across multiple strains and serocomplexes (34–37). Detailed reviews on SS, TS, and CR mAbs against DENV and ZIKV are available in previous studies (36–39).

## QUATERNARY EPITOPES ON FLAVIVIRUSES

With many studies on the determination of epitopes present on flavivirus surface, it is becoming evident that the most potent mAbs, which are GR or CR in nature, recognize quaternary epitopes that emerge when multiple E proteins assemble on the mature virion surface (40, 41). Quaternary epitopes arise from higher-order organization of E proteins and by interactions between adjacent E monomers within a dimer or across neighboring dimers arranged in the viral icosahedral lattice. Hence, these epitopes are conformation-dependent and cannot be replicated by soluble E protein monomers or isolated domains (42). Although the GR and CR bnAbs predominantly recognize quaternary epitopes, some TS-mAbs also do the same. Examples include 5J7, 1F4, 14C10, and 2D22 that bind to epitopes displaying quaternary structures on the smooth virion surface (38, 40, 43–45). Another quaternary-specific antibody against WNV, CR4345, binds to a discontinuous epitope formed by proteins from two neighboring E proteins. A new class of antibody called EDE-C, exemplified by human mAbs DENV-290 and DENV-115, neutralizes DENV3 virus predominantly by blocking virus attachment to the cell. Their epitopes are located in the middle of the E protein dimer on the smooth surface of virions. Hence, only one Fab molecule is required to bind across the E protomers in a dimer (46).

For DENV and ZIKV, not all GR and CR mAbs potently neutralize heterologous strains or species (47, 48). In the past decade, mAbs that can potently neutralize several strains, serotypes, and species have been discovered. These mAbs are referred to as broadly neutralizing antibodies (bnAbs) against flaviviruses, and these recognize highly conserved, quaternary epitopes on the viral E-protein, allowing them to neutralize multiple flaviviruses (49, 50). This review will focus on descriptions of these potent bnAbs against flaviviruses that target quaternary epitopes.

## bnAbs TARGETING QUATERNARY EPITOPES OF FLAVIVIRUSES

The largest breadth in neutralization activity across flaviviruses has been observed in antibodies that recognize a combination of the fusion loop along with adjacent regions on an E protein dimer (38, 45, 48–54). This is a conserved antigenic surface across multiple flaviviruses, as this region also corresponds to the pr peptide’s attachment site on E-protein (51).

One of the well-characterized bnAbs that bind to this quaternary epitope comprising the fusion loop and the adjacent regions on the E protein is referred to as the E-dimer epitope (EDE) bnAbs. EDE bnAbs bind across and interlock two E monomers, with two Fabs binding per E-dimer, stabilizing the prefusion E-dimer conformation. Crystal structures of EDE bnAbs C8, C10, B7, and A11 show that these bind to an overall similar epitope on the E-protein (41, 42, 55, 56). The EDE bnAbs are further sub-classified as EDE1 and EDE2 bnAbs (42, 55). EDE1 strongly neutralizes both DENV and ZIKV, with bnAb binding being independent of N153 glycosylation on DENV E protein. The EDE1 bnAbs, such as C8 and C10, engage primarily via their light chain and disorder the N150 loop. In contrast, the EDE2 bnAbs potently neutralize DENV, with lower activity against ZIKV, and require interaction with the E-protein’s N153 glycan. EDE2 antibodies, such as B7 and A11, recognize the E-protein primarily via the heavy chain, with the light chain involved only in interacting with the N153 glycan. Despite overlapping epitopes and potent neutralization of DENV and ZIKV by EDE1 bnAbs, differential particle dynamics in DENV and ZIKV have been observed upon interaction with the EDE1-C10 antibody in recent HDX-MS and Cryo-EM studies. C10 binding decreases the overall particle dynamics in the case of ZIKV and stabilizes the virus, whereas high concentrations of C10 increase particle dynamics and cause distortion in DENV (57, 58).

**Fig 2:**
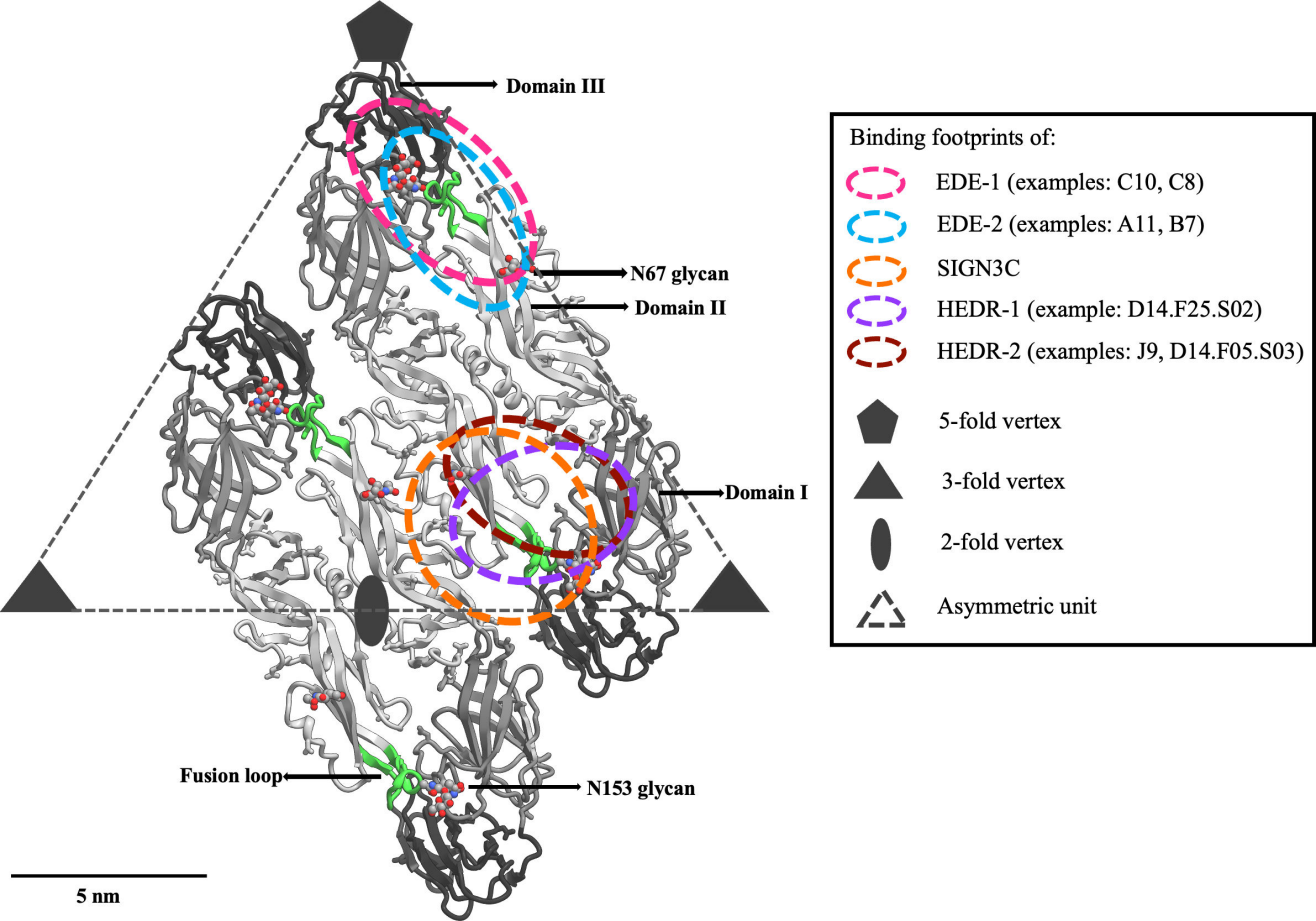
Quaternary epitopes of broadly neutralizing antibodies against flaviviruses. Two parallel envelope protein dimers of one raft have been shown as a ribbon diagram, and the epitope footprints of flavivirus bnAbs are shown as ovals. Domain I, Domain II, Domain III, and the fusion loop are colored gray, light gray, deep gray, and lime green, respectively. The asymmetric unit is shown as a dashed triangle. Scale bar indicates 5 nm.

Another recently characterized class of human antibodies is the Heavy Chain-driven E-dimer recognizing (HEDR) bnAbs that also neutralize all DENV serotypes and, in some cases, ZIKV. HEDR bnAbs bind to an overall similar quaternary epitope as the EDE bnAbs, with the epitope footprint covering the fusion loop region and parts of domain I and domain III from the adjacent E-protein in a dimer (Fig. 2) (27). HEDR bnAbs bind between the conserved glycans, N153 and N67, on the E-dimer. HEDR bnAbs can also be categorized into two types, Category-1 and Category-2. Category-1 HEDR bnAbs, such as D14.F25.S02, neutralize both DENV and ZIKV, with their neutralization activity independent of N153 glycosylation. Category-2 HEDR bnAbs, such as J9, D14.F05.S03, neutralize only DENV serotypes and require N153 glycosylation for their neutralization activity. Despite a globally shared epitope, the specific amino acid requirements for EDE and HEDR bnAbs are distinct, with no distortion of the 150 loop seen on HEDR bnAb binding (27). Furthermore, the HEDR bnAbs preferentially use their heavy chains for the majority of epitope contacts, unlike the EDE bnAbs.

Beyond the quaternary epitope encompassing the fusion loop and intra-E-dimer region described above, bnAbs such as SIgN3C and K8b have also been shown to neutralize several flaviviruses, with the footprints of these bnAbs localized to inter-E-dimer regions (52, 59). SIgN-3C neutralizes all serotypes of DENV and ZIKV and has been shown to have different mechanisms in neutralizing different flaviviruses. It locks surface proteins of DENV at low pH, thereby inhibiting fusion. In ZIKV, it also cross-links the virus particles, causing aggregation and further inhibiting infection (Fig. 2). BnAb K8b is unique in that it has shown broad neutralization against DENV, ZIKV, and also JEV. Structure-based mutagenesis and low-resolution cryo-EM structural analysis show that the bnAb also binds quaternary epitopes with heavy and light chains involved in interaction with domain I, II, and III on E dimers (59).

A characteristic feature of bnAbs recognizing quaternary epitopes is their high neutralizing potency. Notably, EDE and HEDR classes of antibodies demonstrate very low IC50 values (49, 55), which are desirable for combating ADE-related effects. These findings highlight the importance of quaternary epitope-targeting antibodies in neutralization and emphasize the importance of preserving native E-dimer organization in vaccine and immunogen design.

## RECOGNITION OF ALTERNATE VIRION MORPHOLOGIES BY QUATERNARY EPITOPE-TARGETING ANTIBODIES

Some quaternary epitopes recognizing mAbs also bind alternate morphologies of DENV and ZIKV. EDE bnAb, C10, has been reported to bind tubular forms of DENV and ZIKV (26). Similarly, HEDR bnAbs, such as D14.F25.S02, J9, and D14.F05.S03, also bind tubular forms of DENV (27). Another example is the EDE-C bnAb, DENV-290, which also binds to tubular DENV (46). The binding of these bnAbs organizes the tubular regions of DENV in a helical arrangement with the E-dimer as the repeating unit. HmAb 2D22 is an example of a mAb binding to both spherical smooth and bumpy surfaced DENV2 virions (45). In all these examples, the mAb binding epitope is localized to the E-dimer unit, and the binding mechanisms are similar to those observed in the respective wild-type spherical virion complex structures. Nevertheless, such reports are few, requiring further investigations to elucidate how these alternate morphologies affect viral infection and influence ADE.

## IMPLICATIONS FOR VACCINE AND IMMUNOGEN DESIGN

Flaviviruses constitute a major and expanding global public health threat, driven by climate change, urbanization, and global travel. Thus far, there are licensed vaccines available for only five flaviviruses (YFV, DENV, JEV, KFDV, and TBEV). YF-17D (live-attenuated), a licensed vaccine against YFV, is among the most successful of these, as it can induce lifelong immunity and generate strong neutralizing antibody and T cell responses (60, 61). For JEV, JE-VAX, IXIARO (inactivated), and SA14-14-2 (live-attenuated) are licensed and widely used in Asia (62, 63). These vaccines generally induce seroconversion rates of more than 95% within a few weeks and have shown improved safety with predominantly mild, transient, local reactions such as injection site pain and fatigue as side effects. Recently developed chimeric virus (BinJ/JEV _NSW/22_-prME) has also been shown to be a safe and effective vaccine candidate against JEV in a mouse model trial (64). The licensed vaccine for KFDV is a 0.1% formalin-inactivated tissue culture vaccine produced in India (65, 66). Although this vaccine provides partial protection, its limited and waning efficacy, along with the need for frequent booster doses, makes the development of an improved vaccine necessary (67, 68). All licensed vaccines (FSME-IMMUN, Encepur, TBE-Moscow) against TBEV are inactivated whole viruses and are widely used in Europe and parts of Asia. All TBEV vaccines are highly immunogenic with high seroconversion rates ranging from 86% to 100%, although waning of immunity over time has also been reported (69–71).

In the case of DENV, multiple approaches for an effective vaccine are underway. Live-attenuated, chimeric YFV-DENV tetravalent vaccine, CYD-TDV (Dengvaxia), is available but has been reported to increase the risk of severe dengue in seronegative individuals due to ADE, limiting its use (72, 73). Recently, TV003/TV005, a tetravalent live-attenuated vaccine, which is a combination of four recombinant dengue virus vaccine strains (rDEN1D30, rDEN2/4D30, rDEN3D30/31, and rDEN4D30), has demonstrated significant protective efficacy in Phase III clinical trials (74, 75). Recombinant tetravalent vaccines, DEN-80E, TVDV, expressing the prM and E genes of each of the four DENV serotypes from plasmid DNA, have entered phase I clinical trials (76, 77). The tetravalent live-attenuated Takeda dengue vaccine (TAK-003) consists of the live-attenuated DENV-2 PDK-53 strain (TDV-2) as the backbone with the DENV-2 prM and E genes substituted from DENV-116007 (TDV-01), DENV-316562 (TDV-3), and DENV-41036 (TDV-4) (78, 79). It has now been licensed as QDENGA in multiple countries, including Indonesia and European Union member states, although it has not been universally approved (such as the United States). A novel mRNA vaccine encoding the dengue virus serotype 1 envelope and precursor membrane structural proteins (prM/E mRNA-LNP) has been developed and characterized to be delivered via a lipid nanoparticle and has been shown to elicit a protective immune response in mouse models (80). The application of these vaccine candidates has yet to be established in humans, and the observed ADE complications in licensed DENV vaccines till-date emphasize the importance of designing a vaccine that can elicit a balanced response against all DENV serotypes.

Concerns due to ADE-related complications are a primary factor impeding the widespread application of current vaccines against DENV and ZIKV. Current DENV vaccines likely contain structurally heterogeneous particles due to the presence of prM protein in the vaccine designs, which potentially increases the chances of ADE as discussed earlier in this review. Hence, to avoid ADE-related complications, novel immunogen designs that can preferentially display protected epitopes and minimize the exposure of undesirable antigenic sites are needed. Recent structural studies on bnAbs against flaviviruses (54) have established the importance of the E-dimer conformation and E-dimer arrangement as an important factor in broad antigenic recognition. Furthermore, structural studies have shown that potent bnAbs preferentially recognize quaternary epitopes that are present on mature virions, whereas poorly neutralizing antibodies tend to recognize fusion loop epitopes that get exposed in partially mature particles and laboratory-adapted strains (24). Laboratory-adapted strains are dynamic in nature and hence tend to expose fusion loops, which is not the case with clinical strains (21, 22, 25, 44, 45, 81). Hence, antibodies that get elicited against only fusion loop epitopes tend to be non-neutralizing against lesser dynamic clinical strains. Subsequently, stabilized immunogens that limit fusion loop exposure have been explored for DENV serotypes 1–4 by introducing cysteine residues at the E-dimer interface to covalently crosslink E monomers (82, 83). Computational protein design has also been leveraged to construct highly expressing, thermostable E-dimers for DENV serotypes 1–4 and ZIKV to present epitopes similar to the mature virion (84, 85). Immunogen designs that mimic the virus glycoprotein surface have the promise of inducing a better broad neutralizing response than individual domains and peptides. Hence, for designing a subunit vaccine, displaying the native quaternary structure of the viral surface protein, such as stabilizing E protein dimers or multimeric assemblies, has emerged as a promising approach to improve the breadth and potency of protection (86–88).

## CONCLUSION

Quaternary epitopes comprise conserved structural features shared among various flaviviruses. Antibodies targeting these epitopes have shown broader and stronger neutralization, and these epitopes only exist when the E-protein assembles in its native, oligomeric, quaternary form on the infectious virion. Structural studies on bnAbs against flaviviruses have enabled detailed characterization of the bnAb approach and binding features. Further analyses of circulating virus populations can allow us to delineate the occurrence of different morphological forms of flaviviruses. Additionally, analyses of populations with multiple flavivirus infections can aid in isolating a more broadly protective antibody repertoire, paving the way for the development of pan-flavivirus vaccine strategies.
